# Implementation of a Computerized Screening Inventory: Improved Usability Through Iterative Testing and Modification

**DOI:** 10.2196/humanfactors.4896

**Published:** 2016-03-09

**Authors:** Edwin D Boudreaux, Andrew Christopher Fischer, Brianna Lyn Haskins, Zubair Saeed Zafar, Guanling Chen, Sneha A Chinai

**Affiliations:** ^1^ Departments of Emergency Medicine, Psychiatry, and Quantitative Health Sciences University of Massachusetts Medical School Worcester, MA United States; ^2^ Department of Emergency Medicine Kern Medical Center Bakersfield, CA United States; ^3^ Department of Emergency Medicine University of Massachusetts Medical School Worcester, MA United States; ^4^ Department of Internal Medicine Southern Illinois University School of Medicine Springfield, IL United States; ^5^ Department of Computer Science University of Massachusetts Lowell, MA United States

**Keywords:** behavioral medicine, computers, electronic health records, public health, screening, telemedicine

## Abstract

**Background:**

The administration of health screeners in a hospital setting has traditionally required (1) clinicians to ask questions and log answers, which can be time consuming and susceptible to error, or (2) patients to complete paper-and-pencil surveys, which require third-party entry of information into the electronic health record and can be vulnerable to error and misinterpretation. A highly promising method that avoids these limitations and bypasses third-party interpretation is direct entry via a computerized inventory.

**Objective:**

To (1) computerize medical and behavioral health screening for use in general medical settings, (2) optimize patient acceptability and feasibility through iterative usability testing and modification cycles, and (3) examine how age relates to usability.

**Methods:**

A computerized version of 15 screeners, including behavioral health screeners recommended by a National Institutes of Health Office of Behavioral and Social Sciences Research collaborative workgroup, was subjected to systematic usability testing and iterative modification. Consecutive adult, English-speaking patients seeking treatment in an urban emergency department were enrolled. Acceptability was defined as (1) the percentage of eligible patients who agreed to take the assessment (initiation rate) and (2) average satisfaction with the assessment (satisfaction rate). Feasibility was defined as the percentage of the screening items completed by those who initiated the assessment (completion rate). Chi-square tests, analyses of variance, and Pearson correlations were used to detect whether improvements in initiation, satisfaction, and completion rates were seen over time and to examine the relation between age and outcomes.

**Results:**

Of 2157 eligible patients approached, 1280 agreed to complete the screening (initiation rate=59.34%). Statistically significant increases were observed over time in satisfaction (*F*_3,1061_=3.35, *P*=.019) and completion rates (*F*_3,1276_=25.44, *P*<.001). Younger age was associated with greater initiation (initiated, mean [SD], 46.6 [18.7] years; declined: 53.0 [19.5] years, *t*_2,155_=−7.6, *P*<.001), higher completion (*r*=−.20, *P*<.001), and stronger satisfaction (*r*=−.23, *P*<.001).

**Conclusions:**

In a rapid-paced emergency department with a heterogeneous patient population, 59.34% (1280/2157) of all eligible patients initiated the computerized screener with a completion rate reaching over 90%. Usability testing revealed several critical principles for maximizing usability of the computerized medical and behavioral health screeners used in this study. Further work is needed to identify usability issues pertaining to other screeners, racially and ethnically diverse patient groups, and different health care settings.

## Introduction

Electronic health records (EHRs) have become ubiquitous in health care settings, but their full capacity to markedly improve public health has yet to be realized. To catalyze the transition from “public health potential” to “public health improvement,” a landmark collaboration supported by the National Institutes of Health (NIH) Office of Behavioral and Social Sciences Research (OBSSR) has derived a list of recommended core psychosocial screeners to be incorporated into *all* EHRs [1]. The multiphase consensus building process used by the OBSSR collaborative workgroup included diverse stakeholders from health care systems, scientists, policy makers, governmental organizations, health insurers, clinicians, and consumer groups. To facilitate downstream implementation, the panel sought to ensure that the recommended screeners (see Multimedia Appendix 1) were not only rooted in strong science but were also actionable, user friendly, clinically relevant, and cost effective. Adoption of these core measures into EHRs could improve individual-level patient care, identify drivers of hospital readmissions, and facilitate public health research by supporting more efficient, accurate harmonization of data across different EHRs.

Even if an EHR company integrates the core behavioral health screeners into its templates, clinicians still must ask the questions and enter the answers, a task that can be time consuming and susceptible to error, especially when multiple screeners spanning a variety of domains must be administered. Even if paper-and-pencil administration is used by the patient to complete the screeners, the clinician or a designee still must enter the item responses or scale scores into the EHR manually. A highly promising method that avoids these limitations and bypasses third-party interpretation leading to potential misinterpretation is direct entry via computerized assessments. In many situations, electronic collection of screeners is superior to verbal interview because it guarantees standardized administration of the questions and scoring, promotes forthcoming responses by reducing social desirability bias [2-4], and requires less clinician time. Because data from computerized assessments has the potential to be imported directly into the EHR, it can reduce transcription and scoring errors and time associated with manual entry of paper-based item responses or scale scores. A truly integrated system that pairs computerized self-assessments of the OBSSR core screeners such that the data output matches up precisely with the same template fields in the EHR would be a strong innovation. Advances in tablet computing make such integration in clinical settings even more practical, because of the ease of administration, low cost, and growing familiarity with the medium in the general population.

Behavioral health screeners are not the only important information to obtain. From the medical provider’s perspective, computerizing the OBSSR behavioral health screeners alone has limited utility. When managing a heterogeneous group of patients in a general medical setting, like a primary care clinic or an emergency department, screening for medical symptoms, conditions, and diagnoses (eg, pain, chronic illnesses, and surgical history) is equally important. Consequently, for a computerized screening inventory to have optimal utility in general medical settings, it should assist with screening for both medical and behavioral health domains and be integrated with the EHR.

The overall effectiveness of an integrated EHR-computerized screening system assessing both medical and behavioral health statuses will depend on numerous factors. One of the most important is the system’s acceptability and feasibility among patients receiving care. It is essential to design the items and user interface to maximize patient usability. In this context, usability relates to how easy the computerized assessment is to complete [5]. Typically, during usability testing, representative participants are asked to complete the assessment in a manner similar to the intended deployment while trained research staff observe and debrief participants. In addition, data collected by the computerized assessment can be used to evaluate usability, such as examining patterns in missing data to determine challenging items. Usability testing should identify problems that impede successful completion and collect qualitative and quantitative data that help the team to understand the root causes of these impediments. Furthermore, the best usability studies not only identify these impediments but also systematically attempt to remediate them by modifying the items, interface, or administration procedures by evaluating the resulting impact on usability in an iterative fashion.

Although much has been written on designing usable websites from a commercial perspective [6], the literature on usability of computerized screeners designed for use in general medical settings is quite limited. For example, Hess and colleagues [7] published data on more than 11,000 administrations of a tablet-based patient self-assessment in a primary care practice and showed that 84% reported no difficulty in completing the assessment. However, they did not report the proportion of the total population that agreed to complete the computerized assessment, or initiation rate, nor did they obtain systematic information on impediments to completion that may have been used to further improve acceptability and feasibility. While 84% may seem like a strong performance, in busy clinical settings it may be unacceptable, because it suggests that 16% may either be dissatisfied or report problems to clinical staff, who do not have the time or the training to address such issues.

In addition to a general lack of rigorous research on usability of computerized screeners, the association between age and usability remains poorly understood. Some studies have shown age to be inversely associated with usability of computerized assessments [7,8], whereas others have not [9]. The relation between age and usability is important to understand because it could introduce systematic bias into both the clinical monitoring of health behaviors among patients and the public health research that utilizes data resulting from these assessments. Research is needed to better understand how age relates to computerized screener acceptance and feasibility.

The aims of the current study were to (1) computerize a core set of medical and behavioral health screeners, (2) optimize patient acceptability and feasibility through iterative usability testing and modification cycles with a sample of heterogeneous medical patients, and (3) examine how age is associated with patient acceptance and feasibility.

## Methods

### Study Setting

The study was set in a large, urban, academic emergency department, which is a good setting for usability testing of a computerized screening inventory for several reasons. First, because of the nature of emergency care, providers know little about the patients when they arrive. Screening for pain and other past medical history is important.

Second, broad mandates to incorporate behavioral health screening efforts into emergency care exist [10]. This is true for the following reasons: (1) many patients do not have access to primary care, so if behavioral health is not addressed in the emergency department it is often not addressed at all, and (2) many presentations are directly related to health behaviors, such as an automobile crash resulting from driving while intoxicated. Consequently, the emergency department is an important setting in its own right for preventive health efforts. The OBSSR screeners are a particularly good fit for the emergency department because they are very brief, with only 1 or 2 items per screener.

Third, patient volume is brisk and large samples needed for iterative cycles can be generated quickly. The nature of emergency department care allows for patients to have downtime to complete the assessment while they wait for clinician evaluation, test results, consultants, or inpatient beds.

### Participants

From January to December 2013, data collection shifts represented 7 days of the week and ranged between 9 am and 10 pm. During each research shift, every patient who presented for care in the emergency department was logged and considered for participation regardless of presenting complaint to maximize sample representativeness. Patients were excluded if they were younger than 18 years of age, non-English speaking, incarcerated, or medically, cognitively, or emotionally unable to be interviewed or to respond to a computerized assessment (eg, intubation, persistent vomiting, severe pain, altered mental status). Of the 5000 patients logged, 2592 (51.84%) were interviewed by research assistants (RAs); the others were not interviewed due to exclusion criteria (see the “Study Procedure” section), patient unavailability, or research staff unavailability. Of those interviewed, 2157 (83.22%) were deemed eligible; of these, 1280 (59.34%) agreed to take the assessment. The mean (SD) age of the consenting sample was 46 (17) years, and 555 (43.35%) were women, 1021 (79.77%) were white, and 60 (4.69%) were Hispanic.

### Study Procedure

A multidisciplinary team composed of a health psychologist, physicians, nurses, a nurse practitioner, and computer scientists helped build the initial specifications for the computerized screening inventory. The inventory (Vecna Technologies, Inc, Cambridge, MA, USA) is Web-based, hosted on a server compliant with the Health Information Portability and Accountability Act, and designed to be presented on a tablet. The project team created medical screening items that were deemed most important to the emergency department setting. These included pain (intensity and location), other medical symptoms associated with pain, and past medical, psychiatric, and surgical history of the patients (see Multimedia Appendix 1). The OBSSR behavioral health screeners were included, as well as follow-up items in response to positive screens, where appropriate, such as the type of illicit drugs used if the individual screened positive for use. Longer follow-up screeners, such as the Alcohol Use Disorders Identification Test (AUDIT) [11], are recommended by OBSSR for positive initial screens. However, these longer screeners were not included to preserve the feasibility of the administration. The medical items were presented first because the team thought this would promote perceived relevance of completing a computerized screening because most patients present to the emergency department for medical not behavioral complaints.

For the original deployment, the computerized screening inventory was designed to mimic paper intake forms routinely used in medical settings. Multiple items appeared on the screen, and patients indicated their answers by touching the response options and scrolling down to access the rest of the items on the page. Upon completing their current page, patients tapped the “Next” button, and were presented with the next multi-item page. The project team believed that this would be a highly efficient administration format that aligned with a paper-based process with which patients were already familiar with, thereby improving acceptability. The format of the items’ response options was initially allowed to vary based on the particular item. For example, the response to the tobacco use question was binary (Yes/No), whereas illicit drug use was numeric (the number of days in the past 12 months drugs were used). This aligned with the published OBSSR screeners. Patients could skip items at will, a feature the team believed would respect patient’s autonomy by allowing the individual to skip questions he/she did not want to answer. The assessment administration ended automatically after the final answer was entered.

All items and responses used the same font style and size to maintain consistency. All items and instructions were framed in the second person. Because it is difficult to make adjustments simultaneously across numerous languages, only an English version was tested. The project team intends to translate and test the final version with other groups in future studies. The minimum number of items presented was 37 (see Multimedia Appendix 1); the maximum, counting all branched items, was 41 items. Because some screeners required more than 1 item, there are more items than screeners. The computerized screening inventory was extensively tested by the project team, debugged by the Vecna engineers, and piloted with an initial sample of 20 patients prior to full patient testing. Modifications to the base system resulting from usability testing are described in the “Results” section.

Trained RAs first determined if an individual should be excluded through a combination of medical chart review and brief discussion with the treating clinicians. Those clearly satisfying exclusion criteria, such as patients who were being resuscitated, documented as non-English speakers, incarcerated, or physically incapable of completing the electronic assessment were excluded. The rest were approached at the bedside. Approach and consent were concise to make the experience as naturalistic as possible. Following a brief introduction, the RA asked the patient if he/she was willing to participate in a study that involved answering health-related questions on a tablet. The RA assured the patient that experience with computers or tablets was not necessary, their medical care would not be interrupted or delayed by participation, and they could stop at any time. Interested patients provided verbal consent.

For those who consented, the RA opened the computerized screening inventory on the tablet, provided basic instructions, handed the tablet to the patient, and remained present for the first few demographics items (eg, name, age) to ensure the patient understood how to proceed. After the first few items, the RA left the patient’s bedside to provide privacy but remained nearby in case the patient required assistance or was interrupted for medical care. After the patient completed the computerized screening inventory, he/she reviewed a summary of his/her answers for accuracy, and errors were corrected. The RA concluded by performing a semistructured debriefing interview that assessed perceived barriers, challenges, and suggested improvements to the system. The tablet was housed in a protective case and sanitized after every patient administration for infection control.

The RA documented all questions and problems observed throughout the enrollment process, including results from the debriefing interview, on a patient experience log (described in the “Measures” section). This log was summarized by research staff on a weekly basis and reviewed by the principal investigator and other members of the research team. Recommended system changes were identified, prioritized based on their likely impact on usability, and shared with the vendor. Each update to the computerized screening inventory was debugged and tested by a quality assurance team prior to release. Testing, problem identification, and further modifications continued systematically throughout the study period. In addition to changes to the inventory, problems related to the RA’s introduction and administration procedures were identified and solutions implemented. Although small iterative refinements in the software, item content, and administration procedures were made throughout the study, major clusters of changes occurred at 3 time points, which divided the 12 months into 4 phases (see the “Results” section). 

The study was approved by the UMass Institutional Review Board, in accordance with all applicable regulations, and informed consent was obtained after the nature and possible consequences of the study were explained.

### Measures

Demographics

Age, sex, race (white vs. nonwhite), and ethnicity (Hispanic, non-Hispanic) were documented for all patients approached during the research shifts.

Computerized Screening Inventory

The inventory initially consisted of 41 possible items. The medical items were created through team consensus because a standardized medical screening form suitable for the emergency department could not be identified in the literature. Items and response options of the OBSSR screeners followed Estabrooks and colleagues [1], with 2 exceptions. The single-item alcohol screener, “How many times in the past year have you had “X” or more drinks in a day (where “X” is 5 for men, 4 for women)?” was replaced by the 3-item AUDIT-C [12]. The AUDIT-C has been validated in the emergency department setting, whereas the single-item screener has not yet been. The AUDIT-C has an item to assess binge drinking that is very similar to the single-item OBSSR screener, so the computerized screening inventory covered the OBSSR-recommended screening plus 2 items assessing average weekly consumption.

The second deviation pertained to the stress thermometer. Estabrooks and colleagues [1] referred to a “stress” thermometer but used the word “distress” in the item. The “distress” thermometer has never been validated in an emergency department setting and the study team felt that patients would better understand the word “stress,” so it was used instead.

Usability

All RAs made objective observations of the entire administration of the computerized screening inventory, from the initial opening of the inventory to the debriefing interviews. All observations were documented on the patient experience log. This included those observed directly by the RA and those reported by the patient during debriefing. Detailed descriptions of problems were prepared, including representative case studies for team review. Overall completion rates and item skip patterns were summarized intermittently to complement the patient experience log summaries.

Acceptability

Patient acceptability was measured by 2 indicators. First, the “initiation rate” was defined as the number of patients who agreed to take the survey divided by the number of patients who were eligible. Second, the “satisfaction rate” was an average of 3 items administered at the end of the inventory: (1) assessment length (“much too long,” “a little too long,” “about right,” “a little too short,” and “much too short”), (2) ease of understanding the items (“very difficult,” “somewhat difficult,” “neither difficult nor easy,” “somewhat easy,” and “very easy”), and (3) ease of using the tablet (“very difficult,” “somewhat difficult,” “neither difficult nor easy,” “somewhat easy,” and “very easy”). The ratings were averaged to create an overall satisfaction score, with higher scores reflecting stronger satisfaction (range 0-4).

Feasibility

The operational definition of feasibility was the percentage of the survey that an individual completed, or the completion rate. Completion of a screener was counted only if enough information was provided to accurately determine if the patient was positive or negative for the condition. For multi-item screeners, this meant all items had to be answered. In all, 3 completion rates were derived. The overall completion was defined as, among those patients who agreed to participate, the number of screeners completed divided by 15 (the total possible screeners). Medical completion was defined as, among those patients who agreed to participate, the number of medical screeners completed divided by 9 (total number of medical screeners in the inventory). Behavioral health completion was defined as, among those patients who agreed to participate, the number of behavioral health screeners completed divided by 6. Only the 6 behavioral health screeners administered throughout the entire study were used to maintain a consistent denominator across the study. The completion rates ranged from 0% (for a person who agreed to take the survey but did not complete a single screener) to 100% (for a person who completed all of the screeners).

### Data Analytic Plan

Changes in initiation rate (Yes/No, categorical), average satisfaction (continuous), and completion rates (continuous) over time were examined using chi-square tests and one-way analysis of variance (ANOVA), with phase (defined by major upgrades/changes) as the independent variable. Associations between age and outcomes were examined using ANOVAs, independent samples *t* tests, chi-square tests, and Pearson correlations. All data were analyzed using Statistical Package for the Social Science 22 (IBM, Armonk, NY, USA).

## Results

### Usability Testing and Modifications

Table 1 summarizes the major usability problems noted and the resulting changes in the system and administration procedures that were made. Major modifications to the system or administration procedures occurred at 3 points, which split the study into 4 phases. The first major change, which delineated Phase 1 from Phase 2, updated the user interface to use larger font, bolded key phrases, improved contrast between background and items, provided better space separation between items and responses, and presented fewer questions on the screen to eliminate the need for scrolling. Greater clarity on how to navigate the system was added to the RA instructions and the screens, such as how to access the numeric keypad when an integer was needed for a response. In addition, all primary items, or items that were presented to all individuals and which were not branched based on the response to an earlier item, became required rather than allowing “skipping at will” to improve confidence that items with missing data were intentionally skipped. The second major upgrade, which delineated Phase 2 from Phase 3, included presenting a single item per page (rather than multi-item pages), adding “Do not understand” and “Prefer not to answer” to all required items, optimizing the look and feel for tablet presentation, changing all integer response fields to multiple choice “buttons,” and adding the capability of easily editing the items from a final “Confirmation” screen. The total length was shortened by removing 15 items, leaving a total of 29 items. This included removing 3 of the OBSSR screeners that were judged to be less important for the emergency care setting (7 items assessing diet, exercise, sleep) and 8 items assessing demographics. The final major change, which delineated Phase 3 from Phase 4, included adding instructions to help prevent “double tapping” while the Web page was being refreshed between items, which was resulting in some items being inadvertently skipped.

**Table 1:** Usability impediments and solutions applied.

Problem description		Solution applied
**1. Technical**		
	Disrupted Internet connectivity resulted in “frozen assessments” and lost data	Wi-Fi system upgrades (coincidental to the study).
		Tablets were paired with the Clinical Wi-Fi, rather than the Guest Wi-Fi, to improve reliability.
		Staff members were trained to ensure Wi-Fi connection at the beginning of each shift.
		Staff were trained to avoid opening the computerized screening inventory until it was needed to avoid browser time-outs associated with long dormant times.
**2. Survey content/item structure**		
	Survey length prompted discontinued and interrupted assessments, as well as some patient dissatisfaction	The team chose to remove items that were deemed less relevant for the setting and demographics that would likely be already collected in the electronic health record, thereby shortening the total length (from 41 to 26 primary items).
	Integer responses requiring numeric keypad entry were problematic because of skill/knowledge required for accessing the touch screen numeric keypad	All response options were changed to categorical “buttons” (ie, free-text integer responses were eliminated for all items).
	Some patients had trouble understanding or did not want to answer some items	We added 2 response options to every primary item: “Do not understand” and “Prefer not to answer.”
**3. User interface/layout**		
	Skipped items/missing data resulting from multi-item “form” layout (eg, it was difficult to clearly differentiate between items because they were too close together and were skipped, scrolling down to get to the next items led to the patient inadvertently skipping items because they scrolled past them and did not realize it)	Changed from a multi-item “form” based administration to a single item per page.
		Font maximized for single-item presentation.
		No scrolling required.
		Spacing and color contrast were adjusted to maximize differentiation between the item and response options from the background, the item stem from the response options, and the response options from each other.
		Open-response format where patients could skip questions “at will” changed to requiring an answer prior to proceeding to the next question.
	Users sometimes responded to questions but did not realize that they had “tapped” the wrong response until they reviewed the summary of their responses during the debriefing	A final screen was added that allowed the patient to easily review their answers to all of the items and “Confirm” the answers were correct, or easily go back to an item to edit if needed.
**4. Administration process and instructions**		
	Lack of familiarity with touch screen interface created difficulty while navigating and skipped items	
		Opening instructions were modified to be more specific to training patients to understand the basics of responding on a touch screen, including how to scroll and the importance of waiting after tapping a response to avoid double-tapping.
		The option of using a stylus was provided.
		The option of propping the tablet on a tray table was added to help patients who were having trouble holding the tablet (eg, elderly, frail patients).
	Patients could not complete the survey themselves and requested assistance	Family members or friends accompanying the patient could complete the assessment on their behalf (proxy assessment).
	Assessments were interrupted frequently by medical testing, procedures, visitors, etc	A time out and “pause” feature that closes the browser while saving data and allowing resumption from the item last completed was implemented.

Of the 1280 administrations, 61 (4.77%) had a significant technical problem, primarily Wi-Fi interruption; 238 (18.59%) had a usability issue related to the interface, such as problems scrolling or accessing the numeric keypad, although the vast majority of these issues did not prevent the individual from completing the assessment; 411 (32.11%) were interrupted by medical testing, procedures, visitors, or other reasons; and 162 (12.66%) had a family or friend (proxy) complete the assessment on behalf of the patient.

### Acceptability: Initiation

Of the 2592 emergency department patients approached by research staff, 2157 were deemed eligible. Among those eligible, 877 (40.66%) declined and 1280 agreed to participate, for an overall initiation rate of 59.34%. The initiation rate did not differ statistically over the 4 phases, χ_3_^2^ (N=2157) = 8.69, *P*>.05. Those who initiated mean [SD] the survey (46.6 [18.7] years) were younger, on average, than those who declined (53.0 [19.5] years), *t*_2,155_=−7.6, *P*<.001.

### Acceptability: Satisfaction

A one-way ANOVA revealed statistically different average satisfaction rates between phases, *F*_3,1061_=3.35, *P*=.019, with Tukey post hoc tests revealing that satisfaction (mean [SD]) during Phase 3 (3.10 [0.47]) was significantly higher than that during Phase 2 (2.99 [0.57]). Younger age was associated with stronger satisfaction (*r*=−.23, *P*<.001).

### Feasibility: Completion

**Figure 1:**
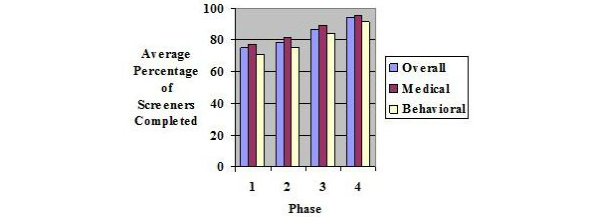
Screener completion rates across the study.

Figure 1 depicts the 3 completion rates (overall, medical, and behavioral) among those who initiated the survey over the 12 months of the study. A one-way ANOVA revealed statistically different average overall completion rates (ie, average percentage of the screeners that were completed) between phases (*F*_3,1276_=25.44, *P*<.001). Tukey post hoc tests revealed that Phase 1 (mean [SD] 75% [38%]) and Phase 2 (79% [35%]) were significantly lower than Phase 3 (87% [30%]), which was, in turn, significantly lower than Phase 4 (94% [19%]). Medical screener completion followed a similar pattern, *F*_3,1276_=23.84, *P*<.001, as did behavioral screener completion, *F*_3,1276_=23.57, *P*<.001. Age was inversely correlated with overall completion (*r*=−.20, *P*<.001), medical screener completion (*r*=−.18, *P*<.001), and behavioral screener completion (*r*=−.20, *P*<.001). The results for each of the screeners, including skip rates, are presented in Multimedia Appendix 2. Multimedia Appendix 2 differs from Multimedia Appendix 1 in that the latter presents all of the individual items administered at the beginning of the study and links them to the screeners with which they are associated, whereas Multimedia Appendix 2 summarizes results pertaining to only 15 screeners that were administered across the entire study.

Overall, 15 screeners were administered. Only the 6 behavioral health screeners that were included throughout the entire study are used to facilitate cross-phase comparison (tobacco, risky alcohol consumption, illicit drug use, depression, anxiety, stress). The phases are defined as follows:

Between Phase 1 and 2=Enlarged text size, bolded key phrases in items, better color contrast between items and background, increased space separation between items and responses, presented fewer questions on the screen, eliminated need for scrolling, greater clarity navigating the system was added to the RA instructions and the computerized screening inventory screens, such as instructions on how to access numeric keypad, all primary questions became required.

Between Phase 2 and 3=Presented a single item per page, added “Do not understand” and “Prefer not to answer” options to all required items, optimized the user interface for tablet presentation, changed all integer response fields to multiple choice buttons obviating need for numeric keypad, added the capability of easily editing the items from the final confirmation screen, shortened by removing 15 items (reduced to a total of 26 primary items).

Between Phase 3 and 4=Added instructions to help prevent “double tapping” while the Web page was being refreshed, which was resulting in some items being inadvertently skipped.

## Discussion

### Preliminary Findings

This is the first study of a computerized screening inventory that blends medical screening items with the NIH OBSSR collaborative’s recommended behavioral health screeners. It is also one of the largest systematic usability studies ever to be conducted on such a system in a general medical setting. The need for systematically testing usability, implementing changes, and testing the effects of these changes was confirmed by the transformative changes that occurred over the course of the study as a result of observations and feedback from patients. These changes were associated with markedly improved completion rates. By the final phase of the study, average overall completion for those who agreed to take the assessment had risen from 75% to exceeding 90%. This result should be interpreted within the demanding context of the setting. Emergency department patients are often uncomfortable, acutely ill, highly heterogeneous, and interrupted frequently due to medical testing and treatment. All of these factors work against survey completion. If rates over 90% can be achieved in the highly demanding emergency department setting, similar or better results can likely be obtained in other more hospitable health care settings, such as primary care. In addition to improved completion rates, overall satisfaction with the computerized screening inventory improved over time, with the biggest improvement observed between Phase 2 and Phase 3 when the survey was shortened and simplified to a single item per page. By contrast, while completion and satisfaction improved, initial agreement to complete the computerized survey remained roughly stable throughout the study at 59.34% (n=1,280/2,157) of all patients who were eligible. The stability of this indicator is not surprising. Other than reassurances that were provided to the patient from the very beginning of the study, such as no computer experience was needed and the assessment would not delay their medical care, strategies to encourage patients to *begin* a computerized assessment are limited. In addition, the acceptance rate may have been suppressed by the fact that this was introduced as a research study, not as part of care. They may have viewed the experience as something that is not essential to their care. If implemented as part of the standardized care, acceptance rates may actually increase.

The lessons learned in this study likely have implications for other applications of computerized screening and assessment, not just those included in this computerized screening inventory, because many of the barriers were nonspecific to the particular items. The most important problems and the associated solutions are reviewed in the following sections.

### Technical Problems

The primary technical barrier centered around the use of Wi-Fi on portable tablets. Tablets are quite popular and are gaining traction in health care settings [13]. Their low cost, portability, and familiarity promote their usability. However, maintaining Internet connectivity when moving from one room to another can be challenging, especially when Wi-Fi capabilities are stressed during peak demand hours and when signals experience interference due to structural barriers. Lost connectivity was the root cause of many of the original “frozen assessments” and lost data. It led to not only entire assessments being lost but also loss of individual questions within an assessment as well. Lost connectivity was made worse by designing the system to avoid caching (temporary storage) of data on the tablets because of data security policies that discourage caching. Interrupted connectivity became less of a problem when the health care system upgraded its Wi-Fi. In addition, research staff training was enhanced. Multiple Wi-Fi networks were available, some with stronger signals than others. RAs were taught how to identify when connectivity to the preferred Wi-Fi had been lost and how to reconnect. Finally, they were trained to log out of the computerized screening inventory completely at the end of the day and to avoid keeping the program open while not in use to avoid browser time outs. Technical solutions that rely on caching, or temporarily storing, data on the hardware and uploading when the connection is restored should also be considered.

### Survey Content

A key challenge that computerized screening can help with is the infeasibility of screening for the plethora of recommended screening domains that exist. While computerization represents a potential solution to this problem, a multidimensional computerized assessment still necessitates more items, which leads to longer administration times. Although many patients tolerated the original 41-item survey quite well, a significant portion were interrupted by medical testing, which made them less likely to complete the assessment. In addition, some patients initially complained it was too long. Even a small percentage of dissatisfied patients can dissuade clinicians from adopting a system like the computerized screening inventory. As a result, the total length was shortened by 37% (n=15/41). There is no optimal length for the number of items a computerized assessment should contain, because it is dependent on a host of factors, some of which relate to the assessment objectives, setting, and population. Tolerance for longer assessments may be better in environments with patients who are not as ill as emergency department patients and care processes that are not characterized by frequent, intermittent medical testing and procedures. Careful testing of the acceptability limits and prioritization of the domains assessed are essential for establishing the optimal length in any setting.

Another important finding pertained to item response formats. The recommended wording and response format for several OBSSR screeners necessitated responding with a free-text integer, such as reporting the number of days one used drugs in the past 12 months. However, entering numeric responses challenged some tablet-naïve patients. It required knowing how to access the numeric keypad, which is not immediately obvious and requires knowledge of the correct button to press to activate it. Consequently, the response format was changed for all items to a categorical, button response modality. For example, the illicit drug item was changed from assessing the number of days in the past 12 months that drugs were used to assessing whether the individual had used any drugs in the past 12 months, Yes/No. This provided for a consistent, categorical response set throughout the assessment, rather than switching back and forth from categorical responses to numeric responses, and avoided any need to access the numeric keyboard, which made completing the assessment easier. Notably, at least four of the OBSSR screeners that use numeric response options (see Multimedia Appendix 1) may need to be adapted when computerized. The impact of this modification on reliability and validity is unknown and may need to be tested prospectively.

### User Interface

Some usability issues were rooted in user interface design choices. Initially, the team sought to design a highly efficient interface that presented multiple items similar to a paper-based form, thereby presenting the information in a format familiar to patients and reducing the number of page turns the individual had to complete. However, this multi-item format was text dense, required smaller font, and the items and responses were spaced too closely together. As a result, patients more easily passed over items, especially while scrolling, or mistakenly selected options near to the intended target. Even when the format changed to remove the need for scrolling yet maintaining the multi-item format by presenting fewer items on the page, some patients, especially those with vision problems, still had difficulty reading the text. Consequently, the interface was ultimately changed to present a single item on a page, which allowed marked improvements in font size and spacing. While this increased the number of page turns needed, it helped to prevent inadvertent skips and promoted accurate response selection.

Because health screenings can assess potentially sensitive topics, like alcohol and drug use, it is important to respect patient autonomy to refuse to answer. Initially, patients could freely skip items if they did not want to respond. However, it was impossible to determine if the missing data were deliberate (the patient did not want to answer the question) or inadvertent (the patient did not see the item). This was addressed by adding 2 response options, “Do not understand” and “Prefer not to answer” to all primary items. This allowed patients to decline to answer an item while still requiring a response to each item, thereby removing the ambiguity around missing data, and helped to flag items that were either poorly worded or potentially sensitive.

The overall item look-and-feel on the page was very important. The design principles that emerged can be summarized as follows: maximize the font size to improve readability, maintain strong differentiation between the item stem and the response options, maintain good spacing between the response options, and allow for the entire response text to be “active” such that touching any part of the response is sufficient to enter a response. These user interface design features are particularly important for visually impaired patients or patients who have fine motor skills impairment that might impede their ability to accurately touch their intended response option. Radio buttons alone, a common response entry method used in computerized surveys, were woefully inadequate.

One additional design feature that is important to highlight is the confirmation process at the end of the assessment. Simply concluding the assessment after the individual completes the final survey item can result in erroneous responses going unnoticed and, ultimately, entered into the individual’s permanent medical record. Incorporating a final screen that allows the patient to review his or her responses and easily edit incorrect values is an important validation step.

### Administration and Instructions

Many screeners, like the OBSSR screeners, are designed for self-administration. However, implementation of computerized screening inventories will have to account for proxy completion, because many users, especially the very ill, elderly, visually impaired, or tablet naïve, preferred to have an accompanying family member or friend complete the assessment for them. To the extent that behavioral health screeners have not been studied for proxy administration, this introduces an unknown source of potential bias in the results. Nevertheless, it clearly improves the usability of the system. Many of the individuals approached would likely not have accepted the offer or completed the assessment if their family or friends had not been allowed to help.

Another practical administration issue that has important design implications pertains to interrupted assessments. In this study, interruptions were frequent, occurring in 32% (n=411/1280) of patients. This was directly related to the nature of care in the emergency department setting, which is characterized by numerous interactions with various health care professionals, medical testing, and treatment procedures. However, interruptions can occur in any medical setting. As a result, computerized assessments require the following features to accommodate interruptions: First, the patient (or proxy) should be able to pause the assessment by clicking a pause button. Second, the system should have a time out feature that saves data and closes the assessment after a period of inactivity. Third, the individual should have the ability to easily resume the assessment from where he or she left off at any point during care.

### Age

Age was inversely associated with initial acceptance, completion, and satisfaction. This creates a cumulative effect of completers being over-represented by younger patients. Hess and colleagues [7] found similar results in primary care. The practical impact of this trend is that alternative methods of gathering the data captured by a computerized assessment will be more commonly used with elderly patients. Allowing proxy completion may partially help adjust for this problem.

### Limitations

The study was set in an emergency department. While this setting is important in its own right for health behavior screening, and there were practical advantages to performing usability testing in this setting, it may have characteristics that can reduce acceptance and feasibility. This includes high patient acuity and frequent interruptions. Thus, further testing of the computerized screening inventory or similar systems in other medical settings is important. The sample may under-represent minority patients. Additional study on the use of computerized screening batteries with nonwhite, non-English speaking patients is needed.

Of the 2157 patients eligible, 877 (40.66%) declined to initiate the assessment. Importantly, the demographics of those who accepted were very similar to those of the general population, with the exception of age (those who initiated were younger). Because of the relatively large sample, the staffing of RA shifts across all 7 days of the week covering morning, afternoon, and evening hours, and the protocols requiring consecutive consideration of all adult emergency department patients, the sample is highly representative of the population from which it was drawn.

The system did not present the screening questions using audio, which might have led to improved completion by those with poor literacy or eyesight. Audio is difficult in the emergency department because of competing noise and difficulty providing headphones for patients in an efficient, infection-controlled manner, which led the team to reject this option for this project.

Some of the wording and response options of the OBSSR screeners were modified from the original publication. This limitation is partially mitigated by the preliminary nature of the original OBSSR recommendations, which were intended to prompt further research such as in this study. In addition, most of the implications for developing a usable behavioral health screening system derived from this study are independent of the specific wording of the items.

For a system like the computerized screening inventory to work clinically, both patients and clinicians will need to embrace it. This study did not test clinician acceptability. The research team wanted to focus on patient usability as the first step and intends to explore clinician acceptability and feasibility next. This is important because there are significant challenges, including EHR integration, data visualization and actionable presentation of results, clinician training, workflow modification, and hardware availability and security.

### Conclusion

This study focused on a single administration of a multi-item, computerized screening inventory that included items developed for emergency medicine by the study team and behavioral screening items collaboratively developed by the NIH OBSSR for wide use. It incorporated sequential phases of evaluation and refinement that allowed statistical comparison to determine whether changes in content, design, functionality, and training actually resulted in improved usability. Study staff members were trained and dedicated to the study and thus, by design, any loss of interest or commitment by clinicians in administering the inventory and documenting problems was countered. Key changes were identified (Table 1) and changes implemented, resulting in improved completion by those who agreed to complete the survey from 75% in Phase 1 to 94% in Phase 4. Satisfaction ratings also improved over time. Future research that integrates this computerized screening inventory with an EHR and assesses clinician acceptability and feasibility is needed. In addition, rigorous testing of this or similar computerized screeners in other settings, including outpatient, inpatient, and specialty medical settings, and in multilingual populations is needed to replicate and extend these findings.

## Appendix

Multimedia Appendix 1 Computerized screeners used in this study.a.

Multimedia Appendix 2 Screener results (N=1280).a.

## Abbreviations

ANOVA: analysis of variance
AUDIT: Alcohol Use Disorders Identification Test
EHR: electronic health record
NIH: National Institutes of Health
OBSSR: Office of Behavioral and Social Sciences Research
RA: research assistant
