# Supplementary material for: Implementation of a Computerized Screening Inventory: Improved Usability Through Iterative Testing and Modification
Source: JMIR Hum Factors. 2016 Mar 9;3(1):e10. doi: 10.2196/humanfactors.4896 (PMC4811667; doi:10.2196/humanfactors.4896)
Supplement: Multimedia Appendix 1 [file humanfactors_v3i1e10_app1.pdf]

Multimedia Appendix 1. Computerized screeners used in this study.[a]

Domain	Item and response options	Items use for screener and associated screener name
**Medical**		
**1. Pain**	1. Please indicate the amount of pain you have been having with your current health problem. 0 to 10, with anchors: 0 (No pain at all), 5 (Moderate pain), 10 (Worse pain ever)	1. Pain
**2. Pain location**	*Branched:* Please check off where you have the worst pain: 1. Head, 2. Neck, 3. Back, 4. Chest, 5. Belly or stomach, 6. Arm, 7. Leg, 8. Hand or fingers, 9. Foot or toes, 10. Genitals, 11. Buttocks, 12. Other location not listed above	
**3. Associated symptoms**	*Branched*: Numerous items branched based on pain location. An associated symptoms item was presented based on pain location. For example, chest pain was followed by an item assessing other symptoms indicative of myocardial infarction, including shortness of breath, dyspnea, radiating pain, etc.	
**4. Pain history**	*Branched:* Have you had pain like this before? 1. Yes, 2. No	
**5. Cardiovascular**	Have you ever had: (CHECK ALL THAT APPLY): 1. Heart disease (coronary artery disease), 2. A heart attack (myocardial infarction), 3. High blood pressure (hypertension), 4. Congestive heart failure (CHF), 5. Poor	2. Cardiovascular disease

Domain	Item and response options	Items use for screener and associated screener name
	circulation in your arms or legs (peripheral vascular disease), 6. Stroke, 12. No, none of these problems	
**6. Pulmonary**	Have you ever had: (CHECK ALL THAT APPLY): 1. Asthma, 2. Chronic obstructive pulmonary disease (COPD) or emphysema, 3. Chronic bronchitis, 4. No, none of these problems	3. Pulmonary disease
**7. Misc chronic**	Have you ever had: (CHECK ALL THAT APPLY): 1. Diabetes, 2. Cancer, 3. High cholesterol, 4. HIV or AIDS, 5. No, none of these problems	4. Other chronic disease
**8. Alcohol addiction history**[b]	Have you ever had a problem with alcohol abuse or addiction? 1.Yes, 2. No	5. Alcohol addiction history
**9. Drug addiction history**[b]	Have you ever had a problem with drug abuse or addiction? 1. Yes, 2. No	6. Drug addiction history
**10. Psyc history**[b]	Have you ever had: (CHECK ALL THAT APPLY): 1. Depression, 2. Bipolar disorder(manic-depression), 3. Anxiety disorder, 4. Panic disorder, 5. Post-traumatic stress disorder, 6. Eating disorder, like anorexia or bulimia, 7. ADHD or ADD, 8. Schizophrenia or schizoaffective disorder, 9. Any other emotional or behavioral disorder, 10.	7. Psychiatric history

Domain	Item and response options	Items use for screener and associated screener name
	No, none of these problems	
**11. Surgical history**	Have you ever had any surgeries? 1. Yes, 2. No	8. Surgical history
**12. Surgery type**	*Branched*: What type of surgery did you have? (CHECK ALL THAT APPLY): 1. Gallbladder surgery (cholecystectomy), 2. Appendix surgery or removal (appendectomy), 3. Heart surgery (examples: surgery on your valves or cardiac stents), 4. Belly surgery (examples: bowel resection, gastric bypass), 5. Transplant (examples: kidney or liver), 6. C- section, 7. Other kind of surgery not listed above	
**13. General health**	In general, would you say your health is: 1. Poor, 2. Fair, 3. Good, 4. Very good, 5. Excellent	9. General health rating
**Behavioral**		
**1. Risky alcohol use**	In the past 12 months:	
	A. How often do you have a drink containing alcohol? 1. Never, 2. Less than monthly, 3. Monthly, 4. Weekly (once a week), 5. 2 - 3 times a week, 6. 4-6 times a week, 7. Daily	10. Risky alcohol use
	Branched: B. A standard drink is one 12-ounce can or bottle of beer, 1 glass of wine, 1 cocktail / mixed drink, or 1 shot of	10. Risky alcohol use

Domain	Item and response options	Items use for screener and associated screener name
	liquor. How many standard drinks containing alcohol do you have on a typical day when you are drinking? 1. 1 drink, 2. 2 drinks, 3. 3 drinks, 4. 4 drinks, 5. 5-6 drinks, 6. 7-9 drinks, 7. 10 or more drinks	
	Branched: C. How often do you have 4 or more drinks on one occasion? Responses as in A.	10. Risky alcohol use
**2. Tobacco use**	Have you used tobacco in the last 30 days? 1. Yes, 2. No	11. Tobacco use
	Branched: Check the kind of tobacco you have used in the past 30 days (CHECK ALL THAT APPLY): 1. Cigarettes, 2. Cigars, 3. Smokeless tobacco, like dip, chew, snuff, or snus	
**3. Drug use**	How many times in the past year have you used an illegal drug or used a prescription drug for non-medical reasons? — — —	12. Illicit drug use
**4. Drug type**	*Branched*: What drugs have you used (not for medical purposes)? (CHECK ALL THAT APPLY): 1. Marijuana (pot, weed, hash), 2. Painkillers (Oxycontin, Percocet, Darvocet, Vicadin, Lortab), 3. Cocaine (powder, crack), 4. Heroin, 5. Tranquilizers (downers, benzos, Xanax, Ativan, Valium), 6. Hallucinogens (lysergic acid diethylamide, phencyclidine, Acid, Mushrooms, salvia, Angel dust, wet), 7. Stimulants (amphetamines, speed, uppers, meth, crank), 8. Sedatives	

Domain	Item and response options	Items use for screener and associated screener name
	(barbiturates, Halcion, Amytal, Miltown, Quaaludes), 9. Ecstasy, 10. Cold or cough medicines, 11. Inhalants (gasoline, glue, paint, paint thinner, nitrous, whippets), 12. Other drug not listed above	
**5. Anxiety**	Over the past 2 weeks, have you been feeling nervous, anxious, or on edge?	13. Anxiety
	Over the past 2 weeks, have you been unable to stop or control your worrying?	13. Anxiety
	Scale: Not at all, Several days, More days than not, Nearly every day	
**6. Depression**	Over the past 2 weeks, have you been feeling down, depressed, or hopeless?	14. Depression
	Over the past 2 weeks, have you had little interest or pleasure in doing things?	14. Depression
	Scale: Not at all, Several days, More days than not, Nearly every day	
**7. Stress**	Please click on the number (0-10) that best describes how much **stress** you have been experiencing. 0 to 10, with anchors: 0 (No stress at all), 5 (Moderate stress), 10 (Extreme stress)	15. Stress
**8. Sleep**	Do you snore or has anyone told you that you snore?	16. Sleep disturbance

**Domain**	**Item and response options**	**Items use for screener and associated screener name**
	1. Yes, 2. No	
	In the past 7 days, I was sleepy during the daytime: 1. Never, 2. Rarely, 3. Sometimes, 4. Often, 5. Always	16. Sleep disturbance
**9. Eating patterns**	Over the past 7 days, how many times a week did you eat fast food or snacks or pizza? __ __	17. Unhealthy eating
	Over the past 7 days, how many servings of fruits/vegetables did you eat each day? __ __	17. Unhealthy eating
	Over the past 7 days, how many soda and sugar sweetened drinks (regular, not diet) did you drink each day?__ __	17. Unhealthy eating
**10. Physical activity**	How many days of moderate to strenuous exercise, like a brisk walk, did you do in the last 7 days? __	18. Exercise
	On those days that you engage in moderate to strenuous exercise, how many minutes, on average, do you exercise at this level? __ __ __	18. Exercise
**Demographics**		
**1. Sex**	Sex	
**2. Race**	What race do you consider yourself?	
**3. Ethnicity**	Do you consider yourself Hispanic or Latino/Latina?	
**4. English speaking**	How well do you speak English?	
**5. Occupation**	What is your current occupational status?	

Domain	Item and response options	Items use for screener and associated screener name
**6. Marital status**	What is your marital status?	
**7. Education**	What is the highest grade or level of schooling you completed?	
**8. Insurance**	What type of insurance do you have?	
**9. Veteran**	Have you ever served on active duty in the armed forces of the U.S.?	

[a] Some screeners consisted of more than 1 item. The table identifies the domain of measurement, the items, and the screener to which the item belongs.

[b]The project team believed it was important to include these behavioral health history questions to capture a broader history than is evident from the OBSSR screeners, which predominately focus on recent events. In addition, the project team included them under "medical screeners" because they are not standardized and were conceptualized as part of the overall medical history.
