# Supplementary material for: Implementation of a Computerized Screening Inventory: Improved Usability Through Iterative Testing and Modification
Source: JMIR Hum Factors. 2016 Mar 9;3(1):e10. doi: 10.2196/humanfactors.4896 (PMC4811667; doi:10.2196/humanfactors.4896)
Supplement: Multimedia Appendix 2 [file humanfactors_v3i1e10_app2.pdf]

Multimedia Appendix 2. Screener results (N=1280)[a].

Domain	Results	
**Medical**		
**1. Pain**	Answered (sufficient to yield screen score):	1147 (89.6%)
	Positive for pain (>0):	979 (85.4%)
	Negative for pain:	168 (14.6%)
	Not answered (missing):	133 (10.4%)
**2. Cardiovascular**	Answered (sufficient to yield a screen score):	1152 (90.0%)
	Positive for cardiovascular disease:	511 (44.4%)
	Negative for cardiovascular disease:	641 (55.6%)
	Not answered (missing):	128 (10.0%)
**3. Pulmonary**	Answered (sufficient to yield a screen score):	1140 (89.1%)
	Positive for pulmonary disease:	390 (34.2%)
	Negative for pulmonary disease:	750 (65.8%)
	Not answered (missing):	140 (10.9%)
**4. Misc chronic**	Answered (sufficient to yield a screen score):	1141 (89.1%)
	Positive for other chronic disease:	697 (61.1%)
	Negative for other chronic disease:	444 (38.9%)
	Not answered (missing):	139 (10.9%)
**5. Alcohol**	Answered (sufficient to yield a screen score):	1113 (87.0%)
**addiction history**	Positive for problem alcohol history:	132 (11.9%)
	Negative for problem alcohol history:	981 (88.1%)

	Not answered (missing):	167 (13.0%)
**6. Drug addiction**	Answered (sufficient to yield a screen score):	1110 (86.7%)
**history**	Positive for problem drug history:	90 (8.1%)
	Negative for problem drug history:	1020 (91.9%)
	Not answered (missing):	170 (13.3%)
**7. Psyc history**	Answered (sufficient to yield a screen score):	1129 (88.2%)
	Positive for past psychiatric history:	570 (50.5%)
	Negative for past psychiatric history:	559 (49.5%)
	Not answered (missing):	151 (11.8%)
**8. Surgical history**	Answered (sufficient to yield a screen score):	1118 (87.3%)
	Positive for past surgical history:	858 (76.7%)
	Negative for past surgical history:	260 (23.3%)
	Not answered (missing):	162 (12.7%)
**9. General health**	Answered (sufficient to yield a screen score):	1075 (84%)
	Positive for poor/fair general health:	300 (27.9%)
	Negative for poor/fair general health:	775 (72.1%)
	Not answered (missing):	205 (16.0%)
**Behavioral**		
**10. Risky alcohol**	Answered (sufficient to yield screen score):	1087 (84.9%)
**use**	Positive for risky use:	243 (22.3%)
	Negative for risky use:	844 (77.6%)

	Not answered (Missing):	193 (15.1%)
**11. Tobacco use**	Answered:	1102 (86.1%)
	Yes (used tobacco in past 30 days):	306 (27.8%)
	No (did not use in past 30 days):	796 (72.2%)
	Not answered (Missing):	178 (13.9%)
**12. Drug use**	Answered:	1077 (84.1%)
	Yes (used drugs in past 12 months):	113 (10.5%)
	No (did not use in past 12 months):	964 (89.5%)
	Not answered (Missing):	203 (15.9%)
**13. Anxiety**	Answered (sufficient to yield screen score):	1030 (80.5%)
	Positive for anxiety:	212 (20.6%)
	Negative for anxiety:	818 (79.4%)
	Not answered (Missing):	250 (19.5%)
**14. Depression**	Answered (sufficient to yield screen score):	1042 (81.4%)
	Positive for depression:	164 (15.7%)
	Negative for depression:	878 (84.3%)
	Not answered (Missing):	238 (18.6%)
**15. Stress**	Answered:	1068 (83.4%)
	Stress ruler:	Mean (SD) 4.38 (2.76)
	Not answered (Missing)	212 (16.6%)

[a]For brevity, only the primary screeners are shown (ie, branched questions are not summarized), and only the 6 OBSSR screeners administered throughout the entire study are summarized. A registered “skip” was not counted as an answer, since the definition of a completed screen was

that enough information was provided to accurately determine if the patient was positive or negative for the condition.
